# Endothelial to mesenchymal transition contributes to nicotine-induced atherosclerosis

**DOI:** 10.7150/thno.42470

**Published:** 2020-04-06

**Authors:** Wei Qin, Longyin Zhang, Zhange Li, Dan Xiao, Yue Zhang, Haiying Zhang, Justine Nyakango Mokembo, Seth Mikaye Monayo, Nabanit Kumar Jha, Philipp Kopylov, Dmitri Shchekochikhin, Yong Zhang

**Affiliations:** 1 School of Pharmacy, Jining Medical University, Rizhao, Shandong, China; 2 Department of Pharmacology (State-Province Key Laboratories of Biomedicine-Pharmaceutics of China, Key Laboratory of Cardiovascular Research, Ministry of Education), Harbin Medical University, Harbin, Heilongjiang, China; 3 Department of Pharmacy, Sir Run Run Shaw Hospital, School of Medicine, Zhejiang University, Hangzhou, Zhejiang, China; 4 Department of Preventive and Emergency Cardiology, Sechenov First Moscow State Medical University, Moscow, Russian Federation; 5 Institute of Metabolic Disease, Heilongjiang Academy of Medical Science, Harbin, Heilongjiang, China

**Keywords:** nicotine, atherosclerosis, EndMT, α7nAChR, Snail

## Abstract

**Rationale**: Nicotine exposure via cigarette smoking is strongly associated with atherosclerosis. However, the underlying mechanisms remain poorly understood. The current study aimed to identify whether endothelial to mesenchymal transition (EndMT) contributes to nicotine-induced atherosclerosis.

**Methods**: ApoE^-/-^ mice were administered nicotine in their drinking water for 12 weeks. The effects of nicotine on EndMT were determined by immunostaining on aortic root and RNA analysis in aortic intima. *In vitro* nicotine-treated cell model was established on human aortic endothelial cells (HAECs). The effects of nicotine on the expression of EndMT-related markers, ERK1/2 and Snail were quantified by real-time PCR, western blot and immunofluorescent staining.

**Results**: Nicotine treatment resulted in larger atherosclerotic plaques in ApoE^-/-^ mice. The vascular endothelial cells from nicotine-treated mice showed mesenchymal phenotype, indicating EndMT. Moreover, nicotine-induced EndMT process was accompanied by cytoskeleton reorganization and impaired barrier function. The α7 nicotine acetylcholine receptor (α7nAChR) was highly expressed in HAECs and its antagonist could effectively relieve nicotine-induced EndMT and atherosclerotic lesions in mice. Further experiments revealed that ERK1/2 signaling was activated by nicotine, which led to the upregulation of Snail. Blocking ERK1/2 with inhibitor or silencing Snail by small interfering RNA efficiently preserved endothelial phenotype upon nicotine stimulation.

**Conclusion**: Our study provides evidence that EndMT contributes to the pro-atherosclerotic property of nicotine. Nicotine induces EndMT through α7nAChR-ERK1/2-Snail signaling in endothelial cells. EndMT may be a therapeutic target for smoking-related endothelial dysfunction and cardiovascular disease.

## Introduction

Cigarette smoking has been considered as an independent risk factor in the induction and progression of atherosclerosis. Exposure to cigarette smoke leads to various mechanisms predisposing to atherosclerosis, including abnormal vascular growth and vascular inflammation, insulin resistance and dyslipidemia, thrombosis, as well as endothelial dysfunctions 1. Nicotine, one of the major active compounds of cigarette smoke, has been identified as a contributing factor to atherogenesis 2, 3.

Endothelial cell (EC) activation and dysfunction is one of the earliest detectable changes in atherosclerosis 4-6. Endothelial to mesenchymal transition (EndMT) is a process wherein ECs lost the endothelial markers and functions and acquire the mesenchymal markers and functions, including loss of tight junctions, increased secretion of extracellular matrix (ECM) proteins, and increased motility 7. EndMT is first found during the embryonic heart development and now thought to play critical roles in a variety of diseases including atherosclerosis 8. Once induced, EndMT promotes plaque growth by increasing deposition of proatherogenic ECM component fibronectin, along with increasing expression of adhesion molecules ICAM and VCAM, thereby promoting adherence of leukocytes to the vessel wall 9. EndMT also enhances atherosclerotic plaque rupture by altering collagen-MMP balance 10. However, the role of EndMT in nicotine-induced atherosclerosis has not been identified.

The effects of nicotine on cells within the vessel wall are mainly mediated by nicotinic acetylcholine receptors (nAChRs), which are each composed of 5 subunits 1. 17 vertebrate nAChR subunits (α1-α10, β1-β4, δ, γ and ɛ) have been identified, which form homomeric or heteromeric channels 11. α8 subunit is not present in human or mammalian species, but present in avian species such as the chicken^12^. Studies have demonstrated that the effect of nicotine to increase EC migration, proliferation and tube formation are largely mediated by α7nAChR 13. In mice deficient in α7nAChR, the angiogenic effect of nicotine in hindlimb ischemia is abolished 14. Based on these reports, we speculate that α7nAChR may play a role in the mediation of the nicotine-induced EndMT.

Extracellular signal-regulated kinase 1/2 (ERK1/2) are components of the mitogen activated protein kinase (MAPK) and are important messengers for extracellular and intracellular signals 15. It has been documented that ERK1/2 signaling pathway is involved in nicotine-mediated neuroprotection effect 16, cancer cell proliferation 17, and post-injury neointimal formation 18. However, it is unknown whether ERK1/2 signaling is involved in nicotine-induced EndMT process.

Snail, Slug, Zeb1, Zeb2, Twist1 and Twist2 are the nuclear transcription factors that can be activated by intracellular signals and finally promote EndMT^19^. Drugs can initiate or ameliorate EndMT by regulating these factors. For example, aqueous extract of *Psoralea corylifolia L.* inhibits lipopolysaccharide-induced EndMT via inhibiting NF-κB-dependent expression of Snail 20. We speculate that nicotine may initiate EndMT through one or more of these transcription factors.

The goals of this study were as follows: 1) to investigate whether nicotine induces EndMT* in vivo* and *in vitro*; 2) to investigate the roles of α7nAChR in nicotine-induced EndMT; 3) to identify the possible involvement of ERK1/2 signaling and EndMT-related transcription factors in nicotine-induced EndMT.

## Methods

### Materials

Nicotine (N3876) was purchased from Sigma-Aldrich (St. Louis, MO, USA). α-bungarotoxin (α-BTX) was obtained from Tocris Bioscience (Minneapolis, MN, USA). PD98059 (P215) was purchased from Sigma-Aldrich (St. Louis, MO, USA).

### Animal model

The study was approved by the Animal Care and Use Committee of Harbin Medical University and complied with the National Institutes of Health Guide for the Care and Use of Laboratory Animals. Eight-week-old male ApoE^-/-^ mice were purchased from Qingzilan Science and Technology Ltd. (Nanjing, China). Mice were kept under standard animal room conditions with humidity 55% - 60% and temperature 22 ± 1°C. All animals were fed with high fat diet (HFD: 10% lard, 4% milk powder, 2% cholesterol and 0.5% sodium cholate) for 12 weeks to establish atherosclerosis. The mice were randomly divided into 3 groups: control group, nicotine group and nicotine + α-BTX group. Mice in control group drank normal water (pH 6.5-8.5). Mice in nicotine group were administered nicotine in their drinking water at a dose of 100 μg/mL for 12 weeks. α-BTX was used as a pharmacological antagonism of α7nAChR. α-BTX was intraperitoneally injected into the ApoE^-/-^ mice once daily at a dose of 0.05 mg/kg^21^. Mice in nicotine + α-BTX group were administered nicotine and α-BTX at the same time for 12 weeks. Water intake and weight were evaluated regularly to ensure similar conditions in all mice.

The concentrations of nicotine used were based on previous studies. For example, nicotine administration at 100 μg/mL in drinking water yields plasma cotinine levels (an indirect indicator of exposure to nicotine) in the mice similar to those observed in moderate smokers (266 ng/mL)^2^, ^13^, ^22^.

### Histology and immunohistochemistry

The aortic root along with the basal portion of the heart was harvested after 12 weeks and fixed with 4% paraformaldehyde at 4°C for 24 h. Then the frozen tissues embedded in optimal cutting temperature (OCT) compound were sectioned into 7 μm thick sections. Atherosclerotic lesions of the aortic root were observed by hematoxylin-eosin (HE) staining as previously reported^23^, ^24^. For immunostaining, the sections were washed with PBS followed by penetration by 4% Triton X-100 and 1% BSA. After blocked with goat serum at 37°C, the sections were incubated in the primary antibodies overnight and in Alexa Fluor 488- or Alexa Fluor 594-conjugated secondary antibodies (Invitrogen, CA, USA) for 1.5 h at room temperature. The results were acquired using a confocal laser scanning microscope (FV300, Olympus, Japan). Fluorescence intensity of CD31 and α-SMA were measured using Image-Pro Plus software (Media Cybernetics, Bethesda, MD, USA).

### Analysis of vascular lipid deposition

Aortas were carefully excised from the mice and opened along the ventral midline. The aortic samples were stained with Oil Red O by dipping them into 2.45 mmol/L of Oil Red O dissolved in a mixture of chloroform and ethanol at a 1:1 ratio for 20-30 min. The samples were washed in 75% ethanol, and the stained aortas were captured using a digital camera.

### Intimal RNA isolation from aorta

Intimal RNA was isolated from mice to examine specific mRNA expressions in the vascular endothelium as previously reported 25-27. Briefly, mouse aorta was exposed, and the peri-adventitial tissues were removed carefully. After perfusion with saline, whole aorta (ascending aorta, aortic arch and descending aorta) was cut out and transferred to a 60 mm dish containing ice-cold PBS. Next, the tip of an insulin syringe needle was carefully inserted into one end of the aorta to facilitate a quick flush of 1 mL TRIzol reagent (Invitrogen, Carlsbad, CA, USA) through it. The intima eluate was collected into a 1.5-mL tube and prepared for RNA extraction.

### Cell culture and transfection

Human aortic endothelial cells (HAECs) were purchased from ScienCell Research Laboratories (Catalog #6100, Carlsbad, CA, USA) and were cultured in endothelial cell medium (pH 7.4) supplemented with 5% FBS, 1% endothelial cell growth factors, and 1% penicillin/streptomycin. The cells were maintained in 5% CO_2_ and 95% air at 37°C. Average serum nicotine level in moderate smokers is 220 nmol/L and the level can reach 440 nmol/L after consumption of a single cigarette 28. Therefore, HAECs were incubated with 50 or 500 nM nicotine for 48 h in our experiments. α-BTX (1 μM) was used to block α7nAChR in cell experiments. PD98059 (20 μM) was added 1 h before nicotine treatment in the corresponding experiments. For transfection experiments, Snail siRNA obtained from GenePharma Co., Ltd (Suzhou, China) was used to knockdown Snail in HAECs. After 24 h of transfection, the medium was replaced by fresh medium with or without nicotine. After drug treatment, the cells were used for cell morphology observation, immunofluorescent staining and protein/RNA extraction.

### Real-time RT-PCR

RNA was isolated as previously reported 29-31. TRIzol reagent was used to extract total RNA from tissues and cells. After reverse transcription with High-Capacity cDNA Reverse Transcription Kit (Applied Biosystems, Foster City, CA, USA), the levels of mRNAs were determined by SYBR Green I (Applied Biosystems, Foster City, CA, USA) and presented as values of 2^-△△Ct^. GAPDH was used as internal control. The sequences of primers are listed in Table S1.

### Western blot

Western blot analysis was performed as described previously 32-35. The cells were lysed in RIPA buffer containing protease inhibitors and phosphatase inhibitors. After quantified, protein samples of 80 μg were loaded on a 10% or 13% SDS-PAGE gel and transferred to nitrocellulose membranes. The membranes were blocked by 5% non-fat milk dissolved in PBS for 2 h, and then were probed with primary antibodies for 12 h at 4°C. Primary antibodies against CD31 (Rabbit), VE-cadherin (Mouse), α-SMA (Mouse), FSP1 (Rabbit), and Snail (Rabbit) were purchased from Abcam, Inc. (Cambridge, MA, USA). Primary antibodies against p-ERK1/2 (Rabbit) and ERK1/2 (Rabbit) were purchased from Cell Signaling Technology (Danvers, MA, USA). GAPDH (Mouse) was used as an internal control and obtained from Zhongshanjinqiao, Inc. (Beijing, China). After incubated with secondary antibodies, western blot bands were analysed by Odyssey Infrared Imaging System (LI-COR, Lincoln, NE, USA).

### Immunocytochemistry

Immunofluorescent staining was performed as described previously 36, 37. To determine the expression and location of related proteins, immunofluorescent staining was performed on HAECs. The cells were fixed with 4% paraformaldehyde for 30 min at room temperature, followed by penetrated with 0.6% Triton X-100 for 1 h. Then the cells were blocked with goat serum for 1 h at room temperature and probed with primary antibodies against CD31, VE-cadherin, α-SMA and FSP1 overnight at 4°C. On the next day, the cells were incubated with Alexa Fluor-conjugated secondary antibodies (Invitrogen, CA, USA) for 1 h at 37°C. The nuclei were stained with DAPI for 20 min. F-actin staining was performed using FITC-phalloidin (Jiamay Biotech, Beijing, China) according to manufacturer's instructions. Briefly, HAECs were fixed with 4% paraformaldehyde for 10 min and washed by 0.1% Triton X-100 for three times. FITC-phalloidin at a dilution of 1:200 with 5% BSA and 0.1% Triton X-100 was used to stain F-actin for 60 min. Fluorescence was examined under a confocal laser scanning microscope (Nikon 80i, Japan).

### Determination of the permeability of HAECs

HAECs were cultured on transwell polycarbonate filters (pore size 3 μm, filter area 0.33 cm^2^, Costar, Cambridge, MA) until becoming cell monolayers, then the cells were treated by nicotine for 24 h. We replaced the lower compartment medium by 700 μL fresh medium and the upper compartment by 300 μL fresh medium containing BSA/FITC (Beijing Biosynthesis Biotechnology Co. LTD, Beijing, China). After 30 min, 50 μL medium was taken from the lower compartments and upper compartments, respectively. Then the medium was transferred into a 96-well dish to detect fluorescence intensity. The permeability coefficient (Pa) was presented as the formula: Pa = [A]/t × 1/S × V/[L], where [A] represents as the fluorescence intensity of the samples of the lower compartment while [L] represents as the upper, t means the time, S means the filter area and V means the volume of the lower compartment.

### Data Analysis

All data in this study are expressed as mean ± SEM. While statistical comparisons among two groups were performed by two-tailed student's t-test, one-away ANOVA was used for multiple comparisons by GraphPad Prism version 6.0. *P* < 0.05 was considered as statistically significant.

## Results

### Nicotine increases atherosclerotic plaque size in ApoE^-/-^ mice

**Figure 1:**
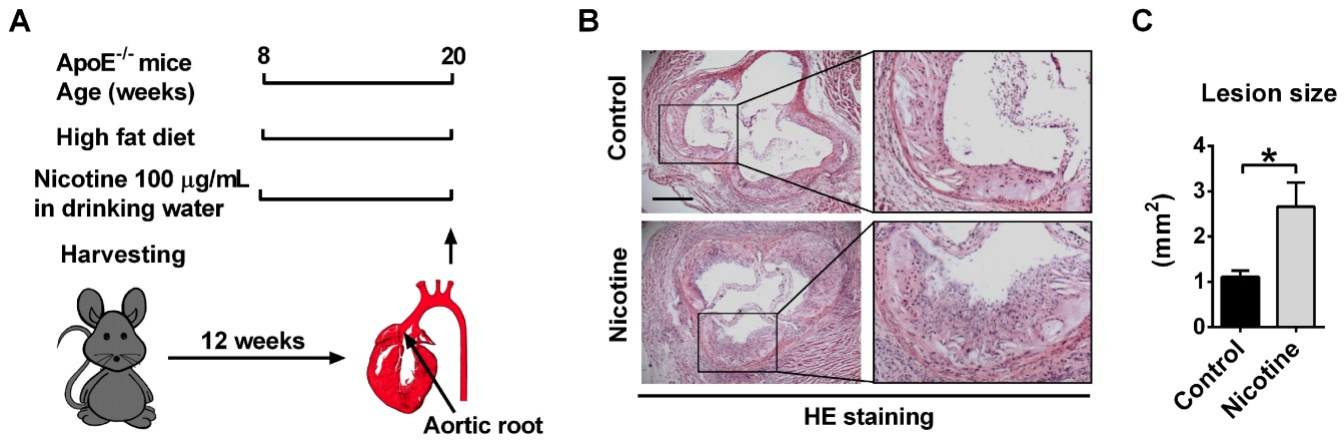
Nicotine exposure promotes atherosclerotic lesions in ApoE^-/-^ mice. (A) Schematic representation of the experimental setup. 8-week-old male ApoE^-/-^ mice were fed with high fat diet (HFD) for 12 weeks to establish atherosclerosis. Mice in control group drank normal water. Mice in nicotine group drank water containing nicotine for 12 weeks. (B) Hematoxylin-eosin (HE) staining of aortic root sections revealing the increase of atherosclerotic lesions induced by nicotine in ApoE^-/-^ mice fed with HFD. Scale bar indicates 600 μm. (C) Quantification of the lesion area per section in the control and nicotine groups. n = 4-6 mice in each group. *P < 0.05.

ApoE^-/-^ mice were used in this study to establish atherosclerotic animal model. We previously showed that 8-week old ApoE^-/-^ mice fed with a high fat diet for 12 weeks displayed typical atherosclerotic plaque not found in those fed with a normal diet^33^. In this experiment, to investigate the role of nicotine in the progression of atherosclerosis, the ApoE^-/-^ mice were divided into two groups: a control group which drinks normal water and a nicotine group which drinks water containing nicotine (Figure 1A). 12 weeks after nicotine administration, HE staining of the aortic sinus was performed. Compared with the control group, nicotine treatment resulted in significantly increased atherosclerotic lesions (control vs. nicotine: 1.11 ± 0.14 vs. 2.66 ± 0.52 mm^2^ ; *P* < 0.05; Figure 1B-C). Moreover, *en face* whole aorta Oil Red O staining revealed increased lipid deposition in aorta in nicotine group compared with control group (Figure S1). These results indicate that nicotine increases atherosclerotic plaque volume in ApoE^-/-^ mice.

### EndMT occurs in the aorta of nicotine-treated ApoE^-/-^ mice

**Figure 2:**
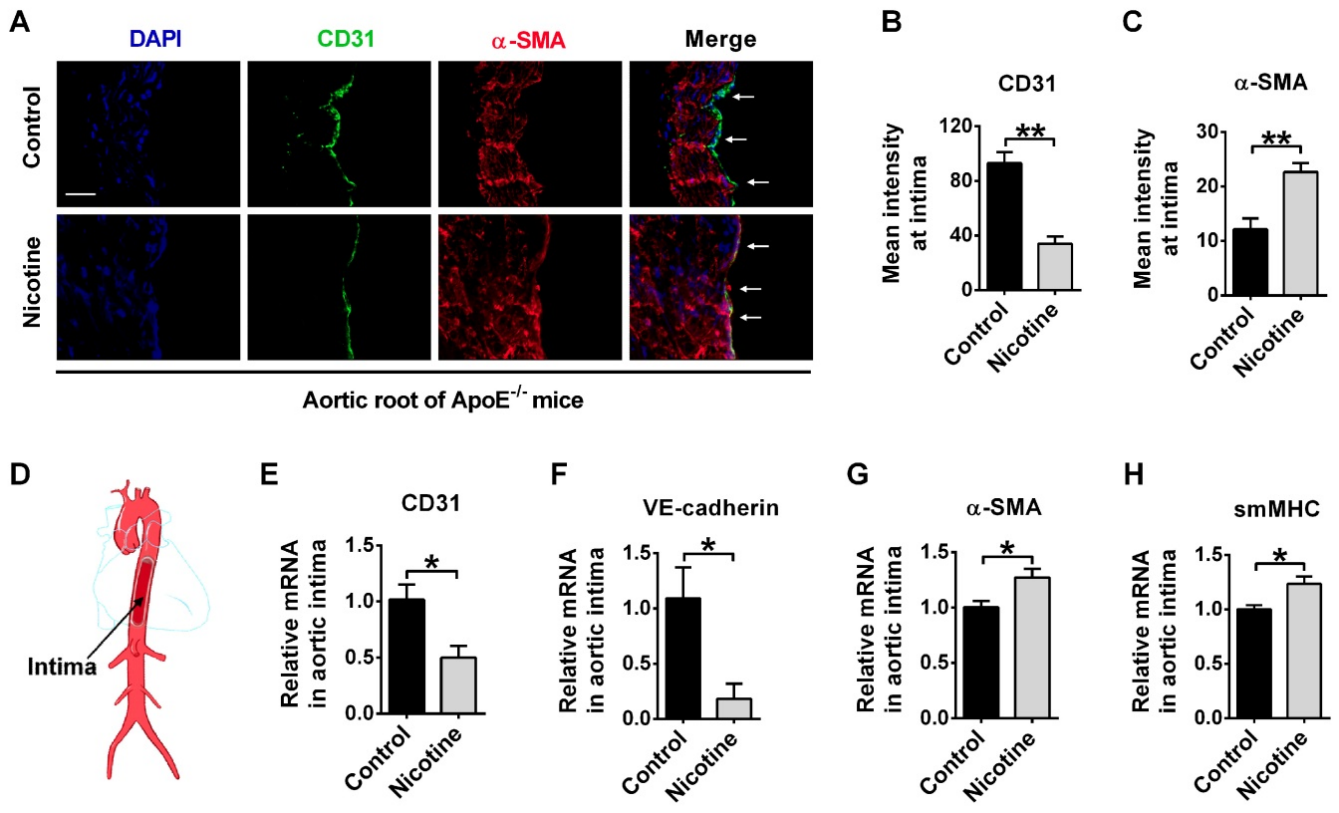
Nicotine triggers endothelial to mesenchymal transition (EndMT) at vascular endothelium in ApoE^-/-^ mice. (A) Representative images of immunofluorescence staining at the intima of aortic root showing CD31 (stained in green) and α-SMA (stained in red) expressions. The nuclei were stained blue with DAPI. Scale bar indicates 50 μm. Arrows indicate differential CD31 and α-SMA expressions in intima endothelial cells. (B-C) Immunofluorescence signals of CD31 and α-SMA were quantified in intima endothelial cells. n = 5 mice in each group. (D) Diagrammatic drawing of the aorta. Intima RNA was harvested for PCR analysis of CD31 (E), VE-cadherin (F), α-SMA (G), and smMHC (H). n = 3-4 mice in each group. *P < 0.05, **P < 0.01.

EndMT has been found to promote atherosclerosis through accumulation of EndMT-derived fibroblast cells in plaques. Therefore, we generated an idea that nicotine may promote atherosclerosis through inducing EndMT. To investigate this, we first tested the expression of EndMT markers in aortic intima using immunofluorescent staining. EndMT is characterized by the degradation of functional endothelial markers like CD31 and VE-cadherin, and the increased expression of mesenchymal-specific markers like α-SMA, smMHC and FSP1^38^. CD31/α-SMA double staining in the aorta showed that CD31 expression was decreased in nicotine group compared with control group. Importantly, cells expressing both CD31 and α-SMA were present in the nicotine group, but such double-positive cells were not detected in control group (Figure 2A). The summarized data of fluorescence signal was shown in Figure 2B-C. To gather further evidence for the acquisition of a mesenchymal phenotype of endothelial cells, aortic intima was harvested for PCR analysis (Figure 2D). We found that CD31 and VE-cadherin were decreased while α-SMA and smMHC were increased in the endothelium of nicotine-treated mice (Figure 2E-H). Taken together, these data demonstrate that EndMT occurs in aortic intima of nicotine-treated ApoE^-/-^ mice.

### Nicotine induces EndMT in HAECs

**Figure 3:**
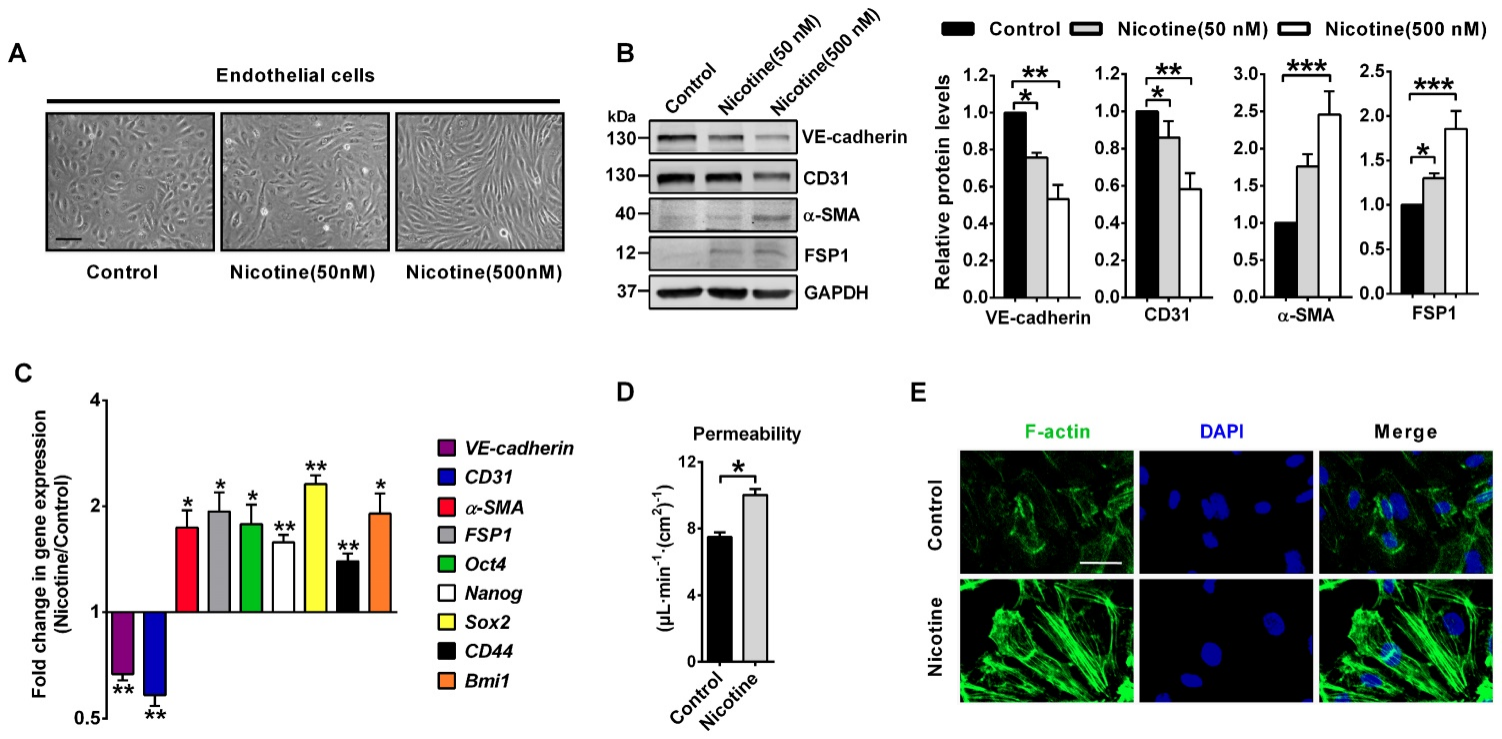
Nicotine triggers endothelial to mesenchymal transition (EndMT) in human aortic endothelial cells (HAECs) *in vitro*. (A) Morphological changes upon nicotine stimulation were observed under the microscope. Scale bar indicates 200 µm. (B) The protein levels of endothelial markers (VE-cadherin and CD31) were downregulated, while mesenchymal markers (α-SMA and FSP1) were upregulated in HAECs after treatment with nicotine, as indicated by western blot. n = 5. (C) The relative mRNA levels of endothelial markers (VE-cadherin and CD31) were downregulated, while mesenchymal markers (α-SMA and FSP1) and stem cell markers (Oct4, Nanog, Sox2, CD44 and Bmi1) were upregulated in HAECs after treatment with nicotine. n = 5. (D) Endothelial permeability was determined using a transwell permeability assay. n = 5. (E) F-actin staining was performed using phalloidin (green) and imaged under the microscope. The nuclei were stained blue with DAPI. Scale bar indicates 50 μm. *P < 0.05, **P < 0.01, ***P < 0.001.

To further confirm the relationship between nicotine and EndMT, we used HAECs for in vitro experiments. In HAECs treated with 50 or 500 nM nicotine, cell morphology changed from round, cobblestone-like endothelial phenotype to elongated, spindle-shaped mesenchymal phenotype (Figure 3A). Western blot and PCR analysis were performed to assess protein and mRNA expression of key molecules involved in EndMT. In HAECs treated with 50 or 500 nM nicotine, endothelial markers CD31 and VE-cadherin were significantly downregulated, while mesenchymal markers α-SMA and FSP1 were upregulated (Figure 3B). Next to that, studies have shown that ECs undergoing EndMT acquire stem cells-like properties 39, so mRNA levels of several stem cell markers, like Oct4, Nanog, Sox2, CD44 and Bmi1 were measured. As shown in Figure 3C, a significant upregulation of these stem cell markers in HAECs was observed upon nicotine stimulation. Results from transwell assays demonstrated that the permeability of HAECs increased significantly after nicotine stimulation (Figure 3D). Furthermore, nicotine-treated HAECs revealed increased formation of actin stress fibers (Figure 3E). It has been well established that induction of EndMT in ECs resulted in a series of abnormal gene expressions which promoted atherosclerotic plaque formation, including the upregulation of leukocyte adhesion molecules (ICAM1 and VCAM1), monocyte chemotactic protein 1 (MCP1), proinflammatory protein plasminogen activator inhibitor-1 (PAI1), matrix metalloproteinases (MMP1, 9 and 10), and TIMP metallopeptidase inhibitors (TIMP2 and 4) and the loss of protective protein endothelial NOS (eNOS)^9^, ^10^. Therefore, mRNA levels of these genes were measured. In HAECs treated with 500 nM nicotine, the expression of ICAM1, VCAM1, MCP1, PAI1, MMP1, MMP9, MMP10, TIMP2 and TIMP4 were significantly upregulated, while eNOS was downregulated (Figure S2). Taken together, these data indicate that nicotine induces EndMT in HAECs. This transition is characterized by a morphological change, a downregulation of vascular endothelial markers, and an upregulation of mesenchymal/stem cell/ pro-atherosclerotic genes and accompanied by cytoskeleton reorganization associated with impaired barrier function.

### Nicotine regulates EndMT through α7nAChR

**Figure 4:**
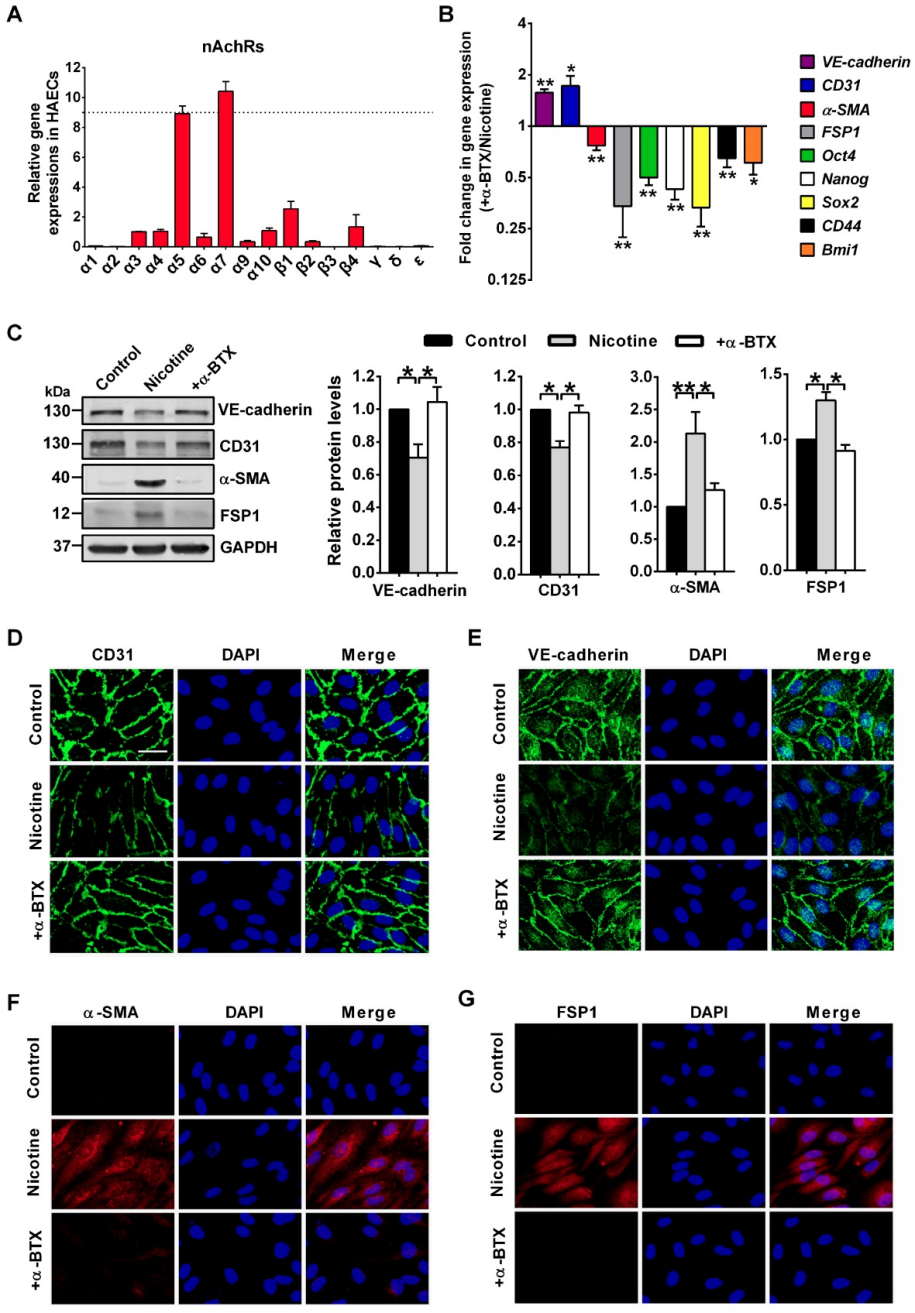
Nicotine triggers endothelial to mesenchymal transition (EndMT) through α7 nicotine acetylcholine receptor (α7nAChR). (A) The relative mRNA levels of 16 nAChR subunits (α1-α7, α9-α10, β1-β4, δ, γ and ɛ) were measured by RT-PCR and normalized to α3 nAChR in human aortic endothelial cells (HAECs). n = 5. (B) The mRNA levels of different EndMT-related markers and stem cell markers were determined by RT-PCR. +α-BTX indicates the co-application of α-BTX with nicotine. n = 5. (C) Protein levels of different EndMT-related markers were determined by western blot. +α-BTX indicates the co-application of α-BTX with nicotine. n = 4. (D-G) Representative images of immunofluorescence staining for EndMT-related markers in HAECs. The nuclei were stained blue with DAPI. +α-BTX indicates the co-application of α-BTX with nicotine. Scale bar indicates 50 μm. *P < 0.05, **P < 0.01.

In this study, we measured the expression of 16 nAChR isoforms (α1-α7, α9-α10, β1-β4, δ, γ and ɛ) in HAECs by PCR analysis. We found α7 isoform showed the highest expression in HAECs (Figure 4A). Several studies have showed that the homomeric α7nAChR mediated the effect of nicotine on endothelial cells 2, 40. Therefore, to identify whether α7nAChR involved in nicotine-induced EndMT, a specific α7nAChR antagonist α-BTX was co-applied with nicotine on HAECs. PCR (Figure 4B), Western blot (Figure 4C), and immunofluorescence (Figure 4D-G) analysis indicated that co-application of α-BTX abolished nicotine-induced VE-cadherin and CD31 downregulation, and reversed nicotine-induced α-SMA and FSP1 upregulation. Furthermore, upon α7nAChR blocking, the levels of stem cell markers Oct4, Nanog, Sox2, CD44 and Bmi1 showed opposite trends compared with nicotine group (Figure 4B). We also found that α-BTX had no significant effect on the expression of EndMT-related markers and stem cell markers in control HAECs (Figure S3). These findings suggest that α7nAChR activation is responsible for nicotine-induced EndMT.

### Blocking α7nAChR relieves nicotine-induced EndMT and atherosclerotic lesions in mice

**Figure 5:**
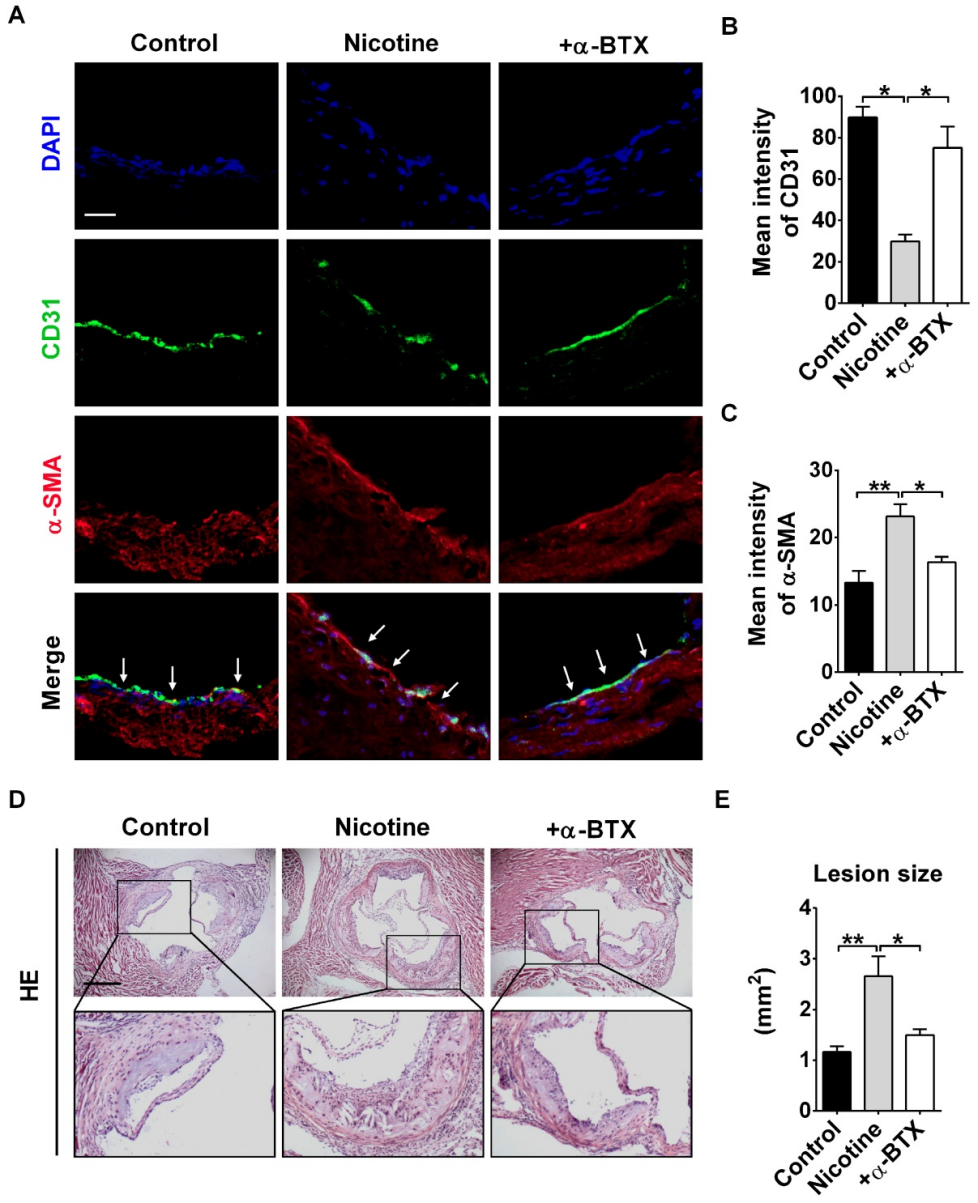
Blocking α7 nicotine acetylcholine receptor (α7nAChR) relieves nicotine-induced endothelial to mesenchymal transition (EndMT) and atherosclerotic lesions in mice. (A) Representative images of immunofluorescence staining at the intima of aortic root showing CD31 (stained in green) and α-SMA (stained in red) expressions. The nuclei were stained blue with DAPI. Scale bar indicates 50 μm. Arrows indicate differential CD31 and α-SMA expressions in intima endothelial cells. +α-BTX indicates the co-administration of α-BTX with nicotine. (B-C) Immunofluorescence signals of CD31 and α-SMA were quantified in intima endothelial cells. +α-BTX indicates the co-administration of α-BTX with nicotine. n = 5 mice in each group. (D) Hematoxylin-eosin (HE) staining of aortic root sections revealing the atherosclerotic lesions in ApoE^-/-^ mice. Scale bar indicates 600 μm. +α-BTX indicates the co-administration of α-BTX with nicotine. (E) Quantification of the lesion area in different groups. +α-BTX indicates the co-administration of α-BTX with nicotine. n = 4-5 mice in each group. *P < 0.05, **P < 0.01.

To further validate the effects of α7nAChR activation on nicotine-induced EndMT and atherosclerotic formation *in vivo*, we administrated α-BTX into mice. Immunofluorescence staining revealed that the expression of endothelial marker CD31 in intima was significantly increased, and CD31^+^α-SMA^+^ cells were barely detectable by α-BTX administration (Figure 5A-C). Additionally, we assessed whether administration of α-BTX could affect nicotine-induced atherosclerotic lesions. As shown in Figure 5D-E, the mice of the α-BTX group displayed decreased atherosclerotic lesion size compared with nicotine group. Administration of α-BTX alone on control ApoE^-/-^ mice exhibited no significant changes in lesion volumes (Figure S4).

### ERK1/2 as central mediators for nicotine-induced EndMT

**Figure 6:**
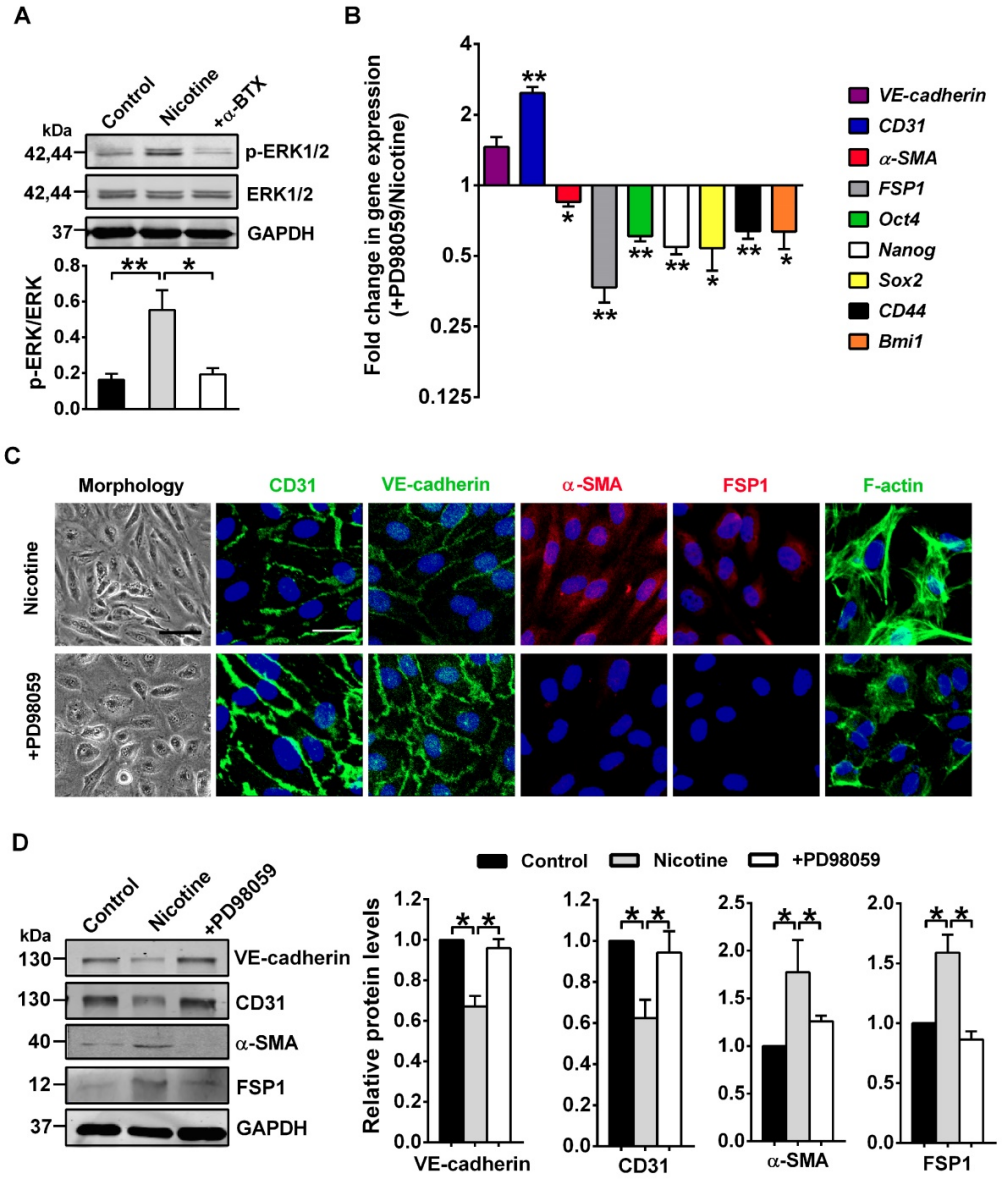
ERK1/2 signaling is involved in nicotine-induced endothelial to mesenchymal transition (EndMT). (A) The protein levels of p-ERK1/2 and total ERK1/2 in human aortic endothelial cells (HAECs) were assessed by western blot. +α-BTX indicates the co-application of α-BTX with nicotine. n = 4. (B) The mRNA levels of different EndMT-related markers and stem cell markers were determined by RT-PCR. +PD98059 indicates the co-application of PD98059 with nicotine. n = 4. (C) Morphological changes, immunofluorescence staining for EndMT-related markers (CD31, VE-cadherin, α-SMA, and FSP1) and F-actin staining were performed. +PD98059 indicates the co-application of PD98059 with nicotine. Scale bar indicates 100 µm (bright field) and 50 µm (fluorescence field). (D) Effects of PD98059 on EndMT-related protein levels. +PD98059 indicates the co-application of PD98059 with nicotine. n = 5. *P < 0.05, **P < 0.01.

ERK1/2, one of the best known conventional MAPK, have been found as key mediators for nicotine-induced pathological processes 41. ERK1/2 also participate in EndMT 42, 43. However, data are lacking on their roles in nicotine-induced EndMT. We hypothesized that ERK1/2 might play a central role in nicotine-induced EndMT. In line with this hypothesis, our data showed a strong induction of ERK1/2 (Thr202/Tyr204) phosphorylation, a necessary step for their activation, upon stimulation of nicotine, and this effect can be abolished by α-BTX treatment(Figure 6A). Furthermore, we pharmacologically inhibited ERK1/2 activation using ERK inhibitor PD98059 44. As illustrated in Figure 6B-D, ERK1/2 inhibition partially reversed the EndMT gene signature induced by nicotine. Also, co-treatment with PD98059 decreased formation of actin stress fibers and changed cell shape to cobblestone-like endothelial phenotype (Figure 6C). Collectively, our data demonstrate a central role for ERK1/2 in inducing EndMT upon nicotine stimulation.

### Nicotine-induced EndMT requires Snail

**Figure 7:**
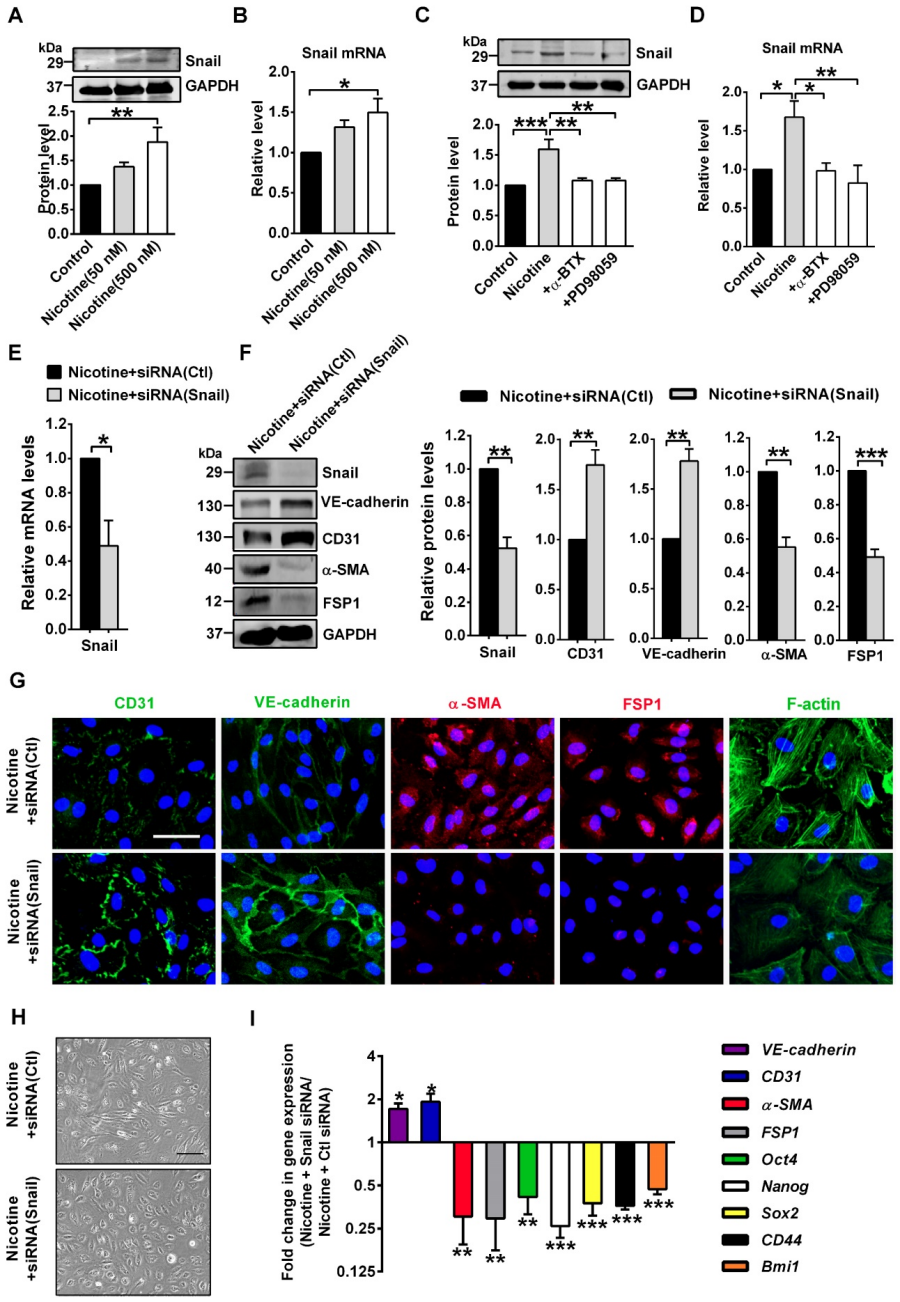
Snail deficiency blocks nicotine-induced endothelial to mesenchymal transition (EndMT). (A) The protein levels of Snail in human aortic endothelial cells (HAECs) were assessed by western blot. n = 4-5. (B) The mRNA levels of Snail were determined by RT-PCR. n = 5. (C-D) Effects of α-BTX and PD98059 on Snail levels in nicotine-treated HAECs. +α-BTX indicates the co-application of α-BTX with nicotine. +PD98059 indicates the co-application of PD98059 with nicotine. n = 3-5. (E) Verification of silencing efficiency of Snail by siRNA in nicotine-treated HAECs. Ctl indicates negative control. n = 5. (F) The protein levels of Snail, CD31, VE-cadherin, α-SMA and FSP1 in nicotine-treated HAECs. n = 5. (G) Immunofluorescence staining for EndMT-related markers and F-actin staining in nicotine-treated HAECs. Scale bar indicates 50 µm. (H) Morphological changes of nicotine-treated HAECs after silencing Snail. Scale bar indicates 200 µm. (I) mRNA levels of different EndMT-related markers and stem cell markers in nicotine-treated HAECs after silencing Snail. n = 5. *P < 0.05, **P < 0.01, ***P < 0.001.

To investigate the possible involvement of transcription factors in nicotine-induced EndMT, we focused on 6 transcription factors from the Snail, Zeb, and Twist families which are required when EndMT occurs, namely Snail, Slug, Zeb1, Zeb2, Twist1 and Twist2 7. PCR analysis indicated that Snail was profoundly upregulated in nicotine-treated HAECs, but the expressions of the other 5 transcription factors were not significantly altered (Figure S5). Therefore, we speculate that nicotine may initiate EndMT through Snail. In HAECs treated with 50 or 500 nM nicotine, Snail protein and mRNA levels were increased significantly (Figure 7A-B). Moreover, α7nAChR or ERK1/2 inhibition weakened the activating effect of nicotine on Snail (Figure 7C-D), indicating Snail may be a downstream effector of ERK1/2 signaling and a key transcription factor responsible for nicotine-induced EndMT. To further characterize the role of Snail in mediating EndMT in nicotine-treated HAECs, we generated a specific Snail siRNA. Treatment of HAECs with Snail siRNA effectively inhibited Snail expression, compared with those treated with negative control siRNA (Figure 7E).

As shown in Figure 7F-I, Snail silencing significantly reversed the EndMT gene signature induced by nicotine, including VE-cadherin, CD31, α-SMA and FSP1, and the stem cell markers Oct4, Nanog, Sox2, CD44 and Bmi1. Snail silencing decreased formation of actin stress fibers (Figure 7G) and changed cell shape to cobblestone-like endothelial phenotype (Figure 7H). Additionally, Snail knockdown significantly reversed the mRNA levels of atherosclerosis-related genes induced by nicotine (Figure S6). These data suggests the importance of Snail in nicotine-induced EndMT.

## Discussion

In the present study, we assessed whether EndMT contributes to nicotine-induced atherosclerosis. Firstly, we showed that oral treatment with nicotine augmented atherogenesis in ApoE^-/-^ mice and induced EndMT in vascular ECs. Secondly, we provided evidence that α7nAChR played a role in nicotine-induced EndMT and blocking α7nAChR effectively relieved this process as well as atherosclerotic lesions in mice. Finally, we found that the ERK1/2-Snail pathway was involved in nicotine-induced EndMT.

Although the number of smokers is decreasing, smoking still accounts for roughly 1 in 7 deaths in the United States. Among the 4000 chemicals in cigarettes, nicotine is considered the contributor to cigarette addiction and diseases. The cigarette sends the nicotine straight to the lungs, where it's absorbed via the alveoli and carried to the organs through blood. It is worth noting that absorption of nicotine across biological membranes depends on pH. Nicotine is a weak base with a p*K*_a_ of 8.0. In acidic environments, such as the smoke from flue-cured tobaccos, found in most cigarettes (pH 5.5-6.0), nicotine is ionized and does not rapidly cross membranes. In alkaline environment, such as the smoke from air-cured tobaccos, found in pipes, cigars, and some European cigarettes (pH 6.5 or higher), nicotine is unionized and well absorbed 45-47. Nicotine is rightly reviled because of its associations with some negative cardiovascular effects, increasing heart rate 48, raising blood pressure 48, causing arteries to constrict 48, promoting myocardial remodeling including hypertrophy and fibrosis 49, increasing risk of ventricular and atrial fibrillation 50, causing endothelial dysfunction 51, inducing dyslipidemia 52, and causing insulin resistance 53. However, there are also opposite reports that nicotine treatment may have beneficial vascular effects like enhancing vascular smooth muscle relaxation 54. Nicotine also shows a good side on its neuroprotective properties in Alzheimer's disease 55 and Parkinson's disease 56. The association of nicotine with atherosclerosis has been recognized for decades. The results in our study and other researchers' have showed that chronic nicotine consumption promoted atherosclerotic plaque development in ApoE^-/-^ mice 57, 58.

An atherosclerotic lesion forms when monocytes adhere to the damaged endothelium, migrate into the sub-endothelium and differentiate into macrophages and foam cells 59, 60. Endothelial dysfunction represents a key step in the initiation and maintenance of atherosclerosis 60. Nicotine can induce endothelial dysfunction including reduced nitric oxide (NO) bioavailability, increased expression of adhesion molecules and even ECs death 51, 58. EndMT was first described by Leonard M Eisenberg in cardiac development, in which ECs lose their endothelial markers e.g., CD31 and VE-cadherin, and acquire mesenchymal markers e.g., α-SMA and FSP1 61. These EndMT-related gene changes were observed in our study in nicotine-treated vascular ECs, both *in vivo* and *in vitro*, indicating nicotine could induce EndMT. The relationship between EndMT and atherosclerosis was found in recent years. Employing endothelial lineage tracking system, the researchers identified that there were approximately 1%-5% EC-derived fibroblast-like cells in atherosclerotic intimal lesions 10. Further bioinformatic analysis of whole-genome sequencing estimated that ECs that undergo EndMT are different from normal ECs as well as mature fibroblasts 10. The EC-derived fibroblast-like cells have the ability to produce the proatherogenic ECM proteins, along with promote adherence of leukocytes 9 and enhance plaque instability 10. Our study unraveled EndMT as a cellular mechanism for the pro-atherosclerotic property of nicotine, thereby expanded our understanding of nicotine-induced atherosclerosis and other cardiovascular diseases.

Another important finding in our study is that nicotine regulates EndMT through α7nAChR. The nAChRs are a group of ligand-gated ion channels mainly expressed in central nervous system. However, recent studies reported their presence in non-neuronal cells, including ECs 62. The α7nAChR was first isolated and sequenced in 1990, and is distinguished from other nAChRs by its low probability of channel opening and rapid desensitization 63. Studies have showed that α7nAChR is one of the most plentiful nAChRs in epithelial cells 64. Our experimental results showed that this is also true in endothelial cells. HAECs incubated with α7nAChR specific inhibitor α-BTX had no nicotine-induced EndMT. Our *in vivo* study also demonstrated that inhibition of α7nAChR suppressed EndMT in response to nicotine treatment. Furthermore, *in vivo* study showed the protective effects of α-BTX on the nicotine-induced atherosclerosis, as evidenced by decreased plaque size. Previously, nicotine was reported to have the pro-atherosclerotic property by activating α7nAChR on mast cells 2. Here, our results provide another interesting mechanism of a critical regulatory effect of α7nAChR on EndMT in nicotine-induced atherosclerosis.

Transforming growth factor β (TGF-β) has a central role in inducing EndMT 65. We first thought that nicotine might induce EndMT by regulating TGF-β expression. However, a study has reported that nicotine stimulation decreased TGF-β1 release in a dose-dependent manner as nicotine concentration increased from 6×10^-8^ M to 6×10^-6^ M, and raising again at the concentration from 6×10^-5^ M to 6×10^-4^ M, but has no modification on TGF-β1 mRNA expression in ECs, which means that nicotine may inhibit TGF-β1 translation or transportation at low concentration but increase those at high concentration 66. In addition, a study by Romani *et al.* also demonstrated that nicotine (from 10^-12^ to 10^-7^ M) reduced TGF-β1 release by ECs 67. In our study, we found that nicotine at low concentration (5×10^-8^ M and 5×10^-7^ M) could induce EndMT, and in these concentrations nicotine decreased TGF-β1 release as reported 66. Therefore, we speculated that nicotine-induced EndMT was not mediated by TGF-β1.

We showed that activation of α7nAChR increased the phosphorylation of ERK1/2. ERK1/2 signaling pathway mediates a range of cell responses to environmental stimuli and is a potent modulator in EndMT upon TGF-β1 stimulation 68. Nicotine activated ERK1/2 signaling in placenta and fetus and eventually inhibits the fetal development 69. Treatment of macrophage with PNU-282987, a selective α7nAChR agonist, effectively inhibited nicotine-induced activation of ERK1/2^70^. Our result supported the notions that activation of α7nAChR is able to activate ERK1/2 signaling. Notably, we also observed that the nicotine-induced EndMT was prevented by a chemical inhibitor of ERK1/2, suggesting that induction of EndMT by nicotine is ERK1/2 signaling-dependent.

We also found ERK1/2 signaling activation increased Snail expression. Snail is an EndMT master regulatory transcription factor and a key mediator of EndMT to various stimuli. Low shear stress generated by blood flow induces dedifferentiation of ECs through EndMT via regulating Snail 71. Snail is also a direct target of HIF1α in hypoxia-induced EndMT^72^. Here we showed that the expression of Snail was increased upon nicotine stimulation and this effect could be prevented by α7nAChR inhibitor and ERK1/2 inhibitor. Moreover, Snail silencing inhibited nicotine-induced EndMT. These results indicate that nicotine induces EndMT at least partially through Snail.

In conclusion, our study provides evidence that EndMT contributes to the pro-atherosclerotic property of nicotine. Nicotine induces EndMT through α7nAChR-ERK1/2-Snail signaling in endothelial cells. Our results indicate that EndMT may be a therapeutic target for smoking-related endothelial dysfunction and cardiovascular disease.

## Supplementary Material

Supplementary figures and table.

## Abbreviations

α7nAChR: α7 nicotine acetylcholine receptor
α-BTX: α-bungarotoxin
EndMT: endothelial to mesenchymal transition
EC: Endothelial cell
ECM: extracellular matrix
ERK1/2: Extracellular signal-regulated kinase 1/2
HE: hematoxylin-eosin
HAECs: human aortic endothelial cells
HFD: high fat diet
MAPK: mitogen activated protein kinase
NO: nitric oxide
nAChRs: nicotinic acetylcholine receptors
OCT: optimal cutting temperature
Pa: permeability coefficient
TGF-β: transforming growth factor β
