# Supplementary material for: Endothelial to mesenchymal transition contributes to nicotine-induced atherosclerosis
Source: Theranostics. 2020 Apr 6;10(12):5276–89. doi: 10.7150/thno.42470 (PMC7196288; doi:10.7150/thno.42470)
Supplement: Supplementary file 1 — Supplementary figures and table. [file thnov10p5276s1.pdf]

# Supplementary material

# Endothelial to mesenchymal transition contributes to nicotine-induced atherosclerosis

Wei Qin, Longyin Zhang, Zhange Li, Dan Xiao, Yue Zhang, Haiying Zhang, Justine Nyakango Mokembo, Seth Mikaye Mokembo, Nabanit Kumar Jha, Philipp Kopylov, Dmitri Shchekochikhin, Yong Zhang

**Supplementary figures 1-6**

**Supplementary table 1**

**Figure S1**

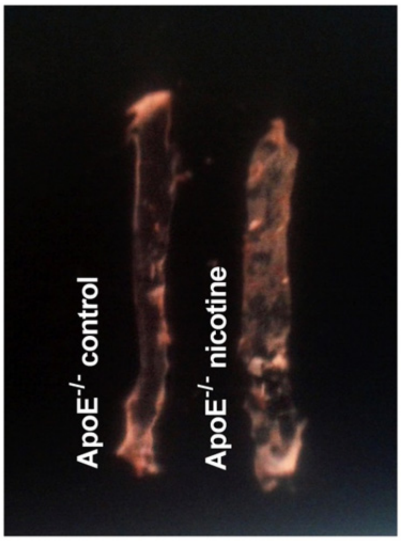


**Figure S1.** Oil Red O staining of aorta reveals the increase of atherosclerotic lesions induced by nicotine in ApoE$^{-/-}$ mice fed with high fat diet.

**Figure S2**

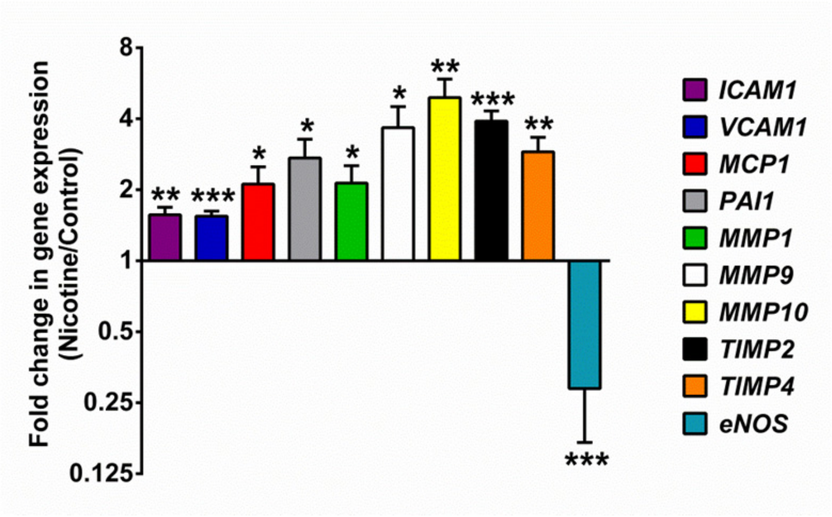


**Figure S2.** Nicotine increases mRNA levels of leukocyte adhesion molecules (ICAM1 and VCAM1), monocyte chemotactic protein 1 (MCP1), proinflammatory protein plasminogen activator inhibitor-1 (PAI1), matrix metalloproteinases (MMP1, 9 and 10), and TIMP metallopeptidase inhibitors (TIMP2 and 4) and decreases mRNA level of protective protein endothelial NOS (eNOS) in human aortic endothelial cells (HAECs). n = 4. *$P < 0.05$, **$P < 0.01$, ***$P < 0.001$.

**Figure S3**

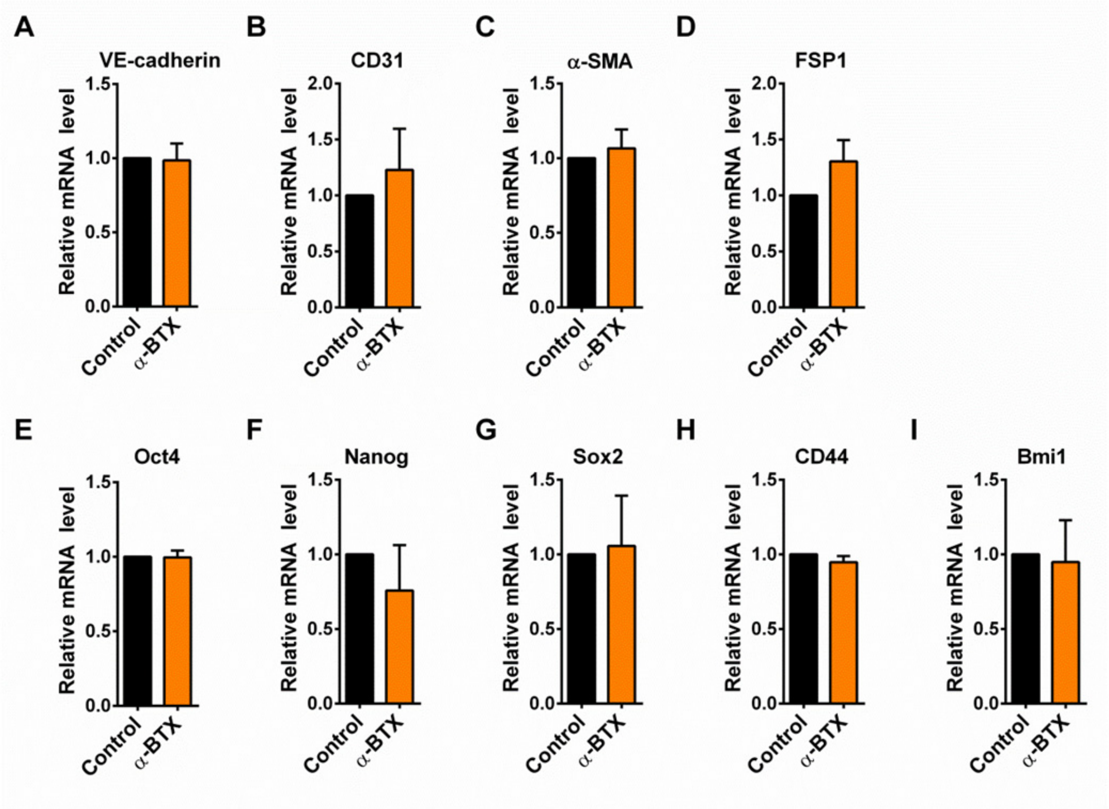


**Figure S3.** Blocking α7 nicotine acetylcholine receptor (α7nAChR) by α-BTX has no obvious effect on the expression of EndMT-related markers and stem cell markers in HAECs. The mRNA levels of VE-cadherin (A), CD31 (B), α-SMA (C), FSP1 (D), Oct4 (E), Nanog (F), Sox2 (G), CD44 (H) and Bmi1 (I) were determined by RT-PCR. n = 4.

**Figure S4**

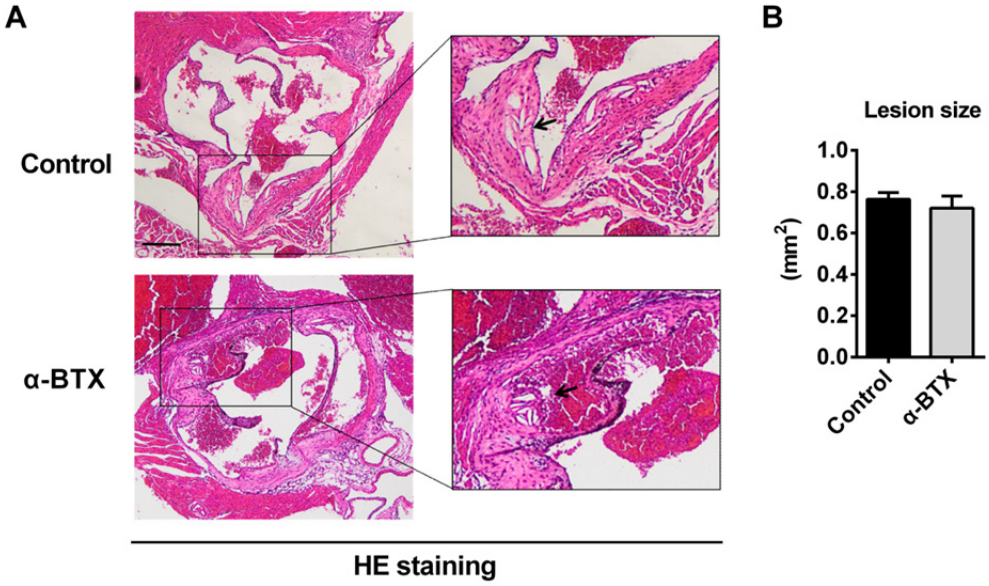


**Figure S4.** Blocking α7 nicotine acetylcholine receptor (α7nAChR) by α-BTX exhibited no significant changes in atherosclerotic lesions in ApoE$^{-/-}$ mice. All animals were fed with high fat diet for 8 weeks to establish atherosclerosis. Mice in α-BTX group received intraperitoneal injection of α-BTX 0.05 mg/kg once daily for 8 weeks. Mice in control group received phosphate buffered saline. (A) Hematoxylin-eosin (HE) staining of aortic root sections. Scale bar indicates 600 μm. Arrows indicate atherosclerotic plaques. (B) Quantification of the lesion area per section in the control and α-BTX groups. n = 4 mice in each group.

**Figure S5**

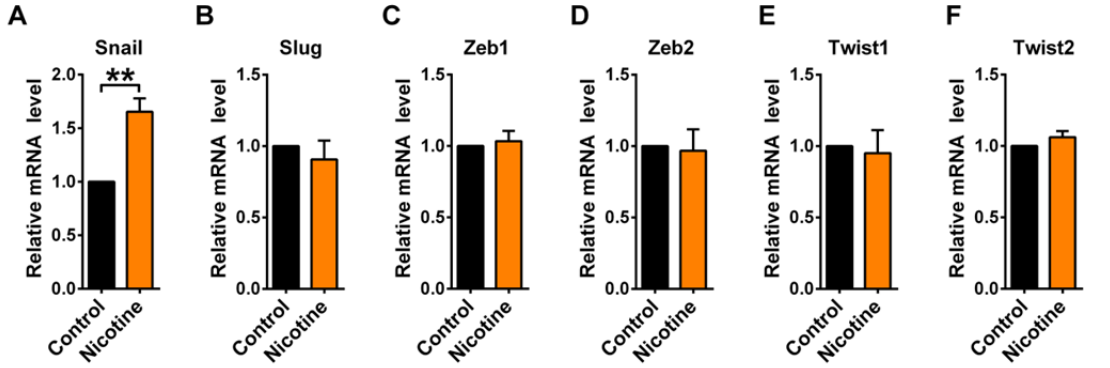


**Figure S5.** Transcription factors Snail was upregulated in HAECs after treatment with nicotine (500 nM). The mRNA levels of Snail (A), Slug (B), Zeb1 (C), Zeb2 (D), Twist1 (E) and Twist2 (F) were determined by RT-PCR. n = 3-4. $^{**}P < 0.01$.

**Figure S6**

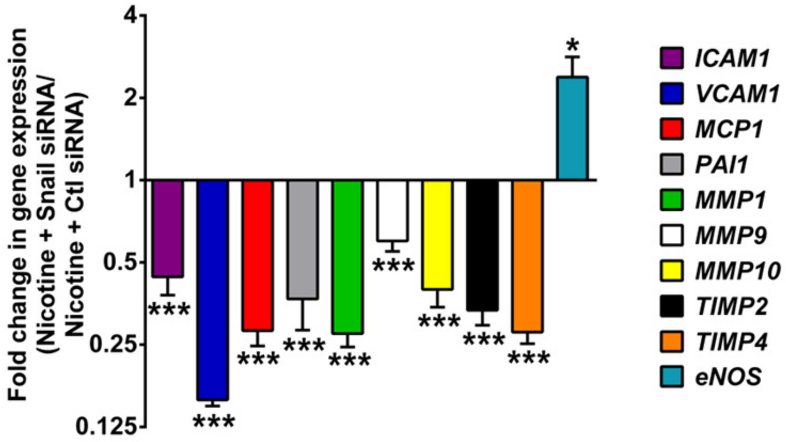


**Figure S6.** Snail knockdown decreases mRNA levels of ICAM1, VCAM1, MCP1, PAI1, MMP1, MMP9, MMP10, TIMP2, and TIMP4 and increases mRNA level of eNOS in nicotine-treated human aortic endothelial cells (HAECs). n = 3-4. *$P < 0.05$, ***$P < 0.001$.

**Table S1. Primers used for qRT-PCR.**

| Gene | Species | Primer Sequence （5’→3’） |
|---|---|---|
| CD31 | Mouse | F ACGCTGGTGCTCTATGCAAG<br>R TCAGTTGCTGCCCATTCATCA |
| VE-cadherin | Mouse | F TCAACGCATCTGTGCCAGAGAT<br>R CACGATTTGGTACAAGACAGTG |
| α-SMA | Mouse | F CCACCGCAAATGCTTCTAAGT<br>R GGCAGGAATGATTTGGAAAGG |
| smMHC | Mouse | F AAGCTGCGGCTAGAGGTCA<br>R CCCTCCCTTTGATGGCTGAG |
| VE-cadherin | Human | F CAGCCCAAAGTGTGTGAGAA<br>R TGTGATGTTGGCCGTGTTAT |
| CD31 | Human | F GAGTCCAGCCGCATATCC<br>R TGACACAATCGTATCTTCCTTC |
| α-SMA | Human | F TGACAATGGCTCTGGGCTCTGTAA<br>R TTCGTCACCCACGTAGCTGTCTTT |
| FSP1 | Human | F GTCCACCTTCCACAAGTAC<br>R TGTCCAAGTTGCTCATCAG |
| Oct4 | Human | F GCAAAGCAGAAACCCTCGTGC<br>R ACCACACTCGGACCACATCCT |
| Nanog | Human | F CAAAGGCAAACAACCCACTT<br>R TCTGCTGGAGGCTGAGGTAT |
| Sox2 | Human | F ATGGGTTCGGTGGTCAAGT<br>R GCTCTGGTAGTGCTGGGACA |
| CD44 | Human | F AAGGTGGAGCAAACACAACC<br>R ACTGCAATGCAAACTGCAAG |
| Bmi1 | Human | F TCCACAAAGCACACACATCA<br>R CTTTCATTGTCTTTTCCGCC |
| α1 nAchR | Human | F GCTCTGTCGTGGCCATCAA<br>R CCGGAAAGCGACCAGCCAGA |
| α2 nAchR | Human | F GTGGAGGAGGAGGACAGA<br>R CTTCTGCATGTGGGGTGATA |
| α3 nAchR | Human | F CAGAGTCCAAAGGCTGCAAG<br>R AGAGAGGGACAGCACAGCAT |
| α4 nAchR | Human | F CTCACCGTCCTTCTGTGTC<br>R CTGGCTTTCTCAGCTTCCAG |
| α5 nAchR | Human | F CTTCACACGCTTCCCAAACT<br>R CTTCAACAACCTCACGGACA |
| α6 nAchR | Human | F TCCATCGTGGTGACTGTGT<br>R AGGCCACCTCATCAGCAG |
| α7 nAchR | Human | F GTACGCTGGTTTCCCTTTGA<br>R CCACTAGGTCCCATTCTC |
| α9 nAchR | Human | F GAAAGCAGCCAGGAACAAAG<br>R GCACTTGGCGATGTACTCAA |
| α10 nAchR | Human | F ACACAAGTGCCCTGAGACCT<br>R TCCCATCGTAGGTAGGCATC |
| β1 nAchR | Human | F CTACGACAGCTCGGAGGTCA<br>R GCAGGTTGAGAACCACGACA |

| | | | |
|---|---|---|---|
| β2 nAchR | Human | F<br>R | GGCATGTACGAGGTGTCCTT<br>CACCTCACTCTTCAGCACCA |
| β3 nAchR | Human | F<br>R | AACAGTTCCGTTTGATTTCACGAT<br>CCCTGATGACCAAGGTCATC |
| β4 nAchR | Human | F<br>R | TCCCTGGTCCTTTTCTTCCT<br>TGCAGCTTGATGGAGATGAG |
| γ nAchR | Human | F<br>R | CGCCTGCTCTATCTCAGTCA<br>GGAGACATTGAGCACAACCA |
| δ nAchR | Human | F<br>R | CAGATCTCCTACTCCTGCAA<br>CCACTGATGTCTTCTCACCA |
| ε nAchR | Human | F<br>R | TCAAGGTCACCCTGACGAAT<br>GTCGATGTCGATCTTGTTGA |
| ICAM1 | Human | F<br>R | CTTTCATTGTCTTTTCCGCC<br>ATGCCCAGACATCTGTGTCC |
| VCAM1 | Human | F<br>R | GGGAAGATGGTCGTGATCCTT<br>TCTGGGGTGGTCTCGATTTTA |
| MCP1 | Human | F<br>R | CAGCCAGATGCAATCAATGCC<br>TGGAATCCTGAACCCACTTCT |
| PAI-1 | Human | F<br>R | ACCGCAACGTGGTTTTCTCA<br>TTGAATCCCATAGCTGCTTGAAT |
| MMP1 | Human | F<br>R | AAAATTACACGCCAGATTTGCC<br>GGTGTGACATTACTCCAGAGTTG |
| MMP9 | Human | F<br>R | TGTACCGCTATGGTTACACTCG<br>GGCAGGGACAGTTGCTTCT |
| MMP10 | Human | F<br>R | TGCTCTGCCTATCCTCTGAGT<br>TCACATCCTTTTCGAGGTTGTAG |
| TIMP2 | Human | F<br>R | AAGCGGTCAGTGAGAAGGAAG<br>GGGGCCGTGTAGATAAACTCTAT |
| TIMP4 | Human | F<br>R | CCACTCGGCACTTGTGATTC<br>CATCCTTGACTTTCTCAAACCCT |
| eNOS | Human | F<br>R | TGATGGCGAAGCGAGTGAAG<br>ACTCATCCATACACAGGACCC |
| Snail | Human | F<br>R | GCCTTCAACTGCAAATACTGC<br>CTTCTTGACATCTGAGTGGGTC |
| Slug | Human | F<br>R | CGAACTGGACACACATACAGTG<br>CTGAGGATCTCTGGTTGTGGT |
| Zeb1 | Human | F<br>R | GATGATGAATGCGAGTCAGATGC<br>ACAGCAGTGTCTTGTTGTTGT |
| Zeb2 | Human | F<br>R | CAAGAGGCGCAAACAAGCC<br>GGTTGGCAATACCGTCATCC |
| Twist1 | Human | F<br>R | TCGGACAAGCTGAGCAAGATT<br>GCAGCTTGCCATCTTGGAGT |
| Twist2 | Human | F<br>R | GGCGCAAGTGGAATTGGGATG<br>CCGGGTCTTCTGTCCGATGT |
| GADPH | Mouse<br>Human | F<br>R | AAGAAGGTGGTGAAGCAGGC<br>TCCACCACCCAGTTGCTGTA |
